# Gender Medicine and Pharmacology

**DOI:** 10.3390/biomedicines12020265

**Published:** 2024-01-24

**Authors:** Sarah Allegra, Francesco Chiara, Silvia De Francia

**Affiliations:** Laboratory of Clinical Pharmacology Service “Franco Ghezzo”, Department of Clinical and Biological Sciences, University of Turin, San Luigi Gonzaga Hospital, 10043 Orbassano, Italy; francesco.chiara@unito.it (F.C.); silvia.defrancia@unito.it (S.D.F.)

Gender-specific medicine consists of a transversal methodological approach that aims to study the influence of sex and gender on diseases. Gender-specific medicine aims to understand how diseases of all organs and systems manifest themselves in the two sexes and, above all, to evaluate the differences in symptoms, the need for different diagnostic paths and interpretations of results, the differences in response to drugs, or even the need to use different drugs. Again, there are differences related to the prevention of all diseases. In fact, treatment response varies between men and women; these differences are mostly due to anatomical, physiological, and hormonal factors. The response to treatments is influenced by variations in the pharmacokinetics and pharmacodynamics of therapeutic agents [1,2,3,4,5,6,7,8,9,10,11,12,13,14,15,16,17,18,19,20,21,22,23,24,25,26,27,28,29,30,31,32,33,34,35,36]. Age and hormones play an important role in drug adsorption, distribution, metabolism, and elimination [37]. Considering females, changes in endogenous sex steroid hormones that occur naturally during the menstrual cycle, during pregnancy, and during the menopausal transition may have an impact on treatment effectiveness and adverse drug reactions [38]. Unlike female reproductive aging (menopause) or organic androgen deficiency in males (due to disorders of the brain, pituitary, or testes), male reproductive aging does not lead to a total cessation of testosterone production or of spermatogenesis. In addition, testosterone insufficiency affects a tiny percentage of aged men and is determined by the existence of comorbidities. Serum testosterone levels are generally normal in older men who maintain good health, and age-related low testosterone has been referred to andropause, viropause, partial androgen deficit in the aging man, and late-onset hypogonadism [39]. Testosterone injection is used to virilize gender-incongruent patients who were given the female gender at birth (AFAB). Even when the same hormone formulations and regimens are utilized, inter-individual variations in the timing and acquisition of phenotypic traits are commonly noted. Pallotti et al. investigated the potential associations between the CpG methylation profile of the H19 and estrogen receptor 2 gene (ESR2) and their impact on phenotypic changes in a group of 13 individuals with AFAB at baseline (T0) and following six months (T6) and twelve months (T12) of testosterone treatment [40]. They speculated that exogenous testosterone treatment may alter the ESR2 methylation pattern. Additionally, they noted that epigenetic modifications seemed to be controlled, implying that the regulation of estrogen signaling is important from the start of the treatment until T6, at which point it ceases to be significantly altered until T12. Moreover, it appears that exogenous testosterone administration and subject age are related to estrogen receptor methylation, as a marker of androgenic therapy. Furthermore, the gonadal axis is inhibited when certain drugs, such as opioids and glucocorticoids, are taken [41,42,43]. Eventually, older men with higher body mass index are more susceptible to oxidative stress, linked to insulin resistance, low sperm concentration, and erectile dysfunction [44]. Considering pharmacodynamics, many variations exist based on sex, mostly caused by hormones, genetics, and environmental factors [28]. Prolactin and female steroid hormones influence immunity. The hypothalamic–pituitary–adrenal and hypothalamic–pituitary–gonadal axes regulate immunity, resulting in two to ten times higher prevalence and severity of inflammatory and autoimmune diseases in females than in males. It is mostly females of reproductive age who suffer from autoimmune diseases. The role of sex is also debated in a number of allergy disorders and their treatment with omalizumab, the first biologic medication approved for use in the field of allergology [45]. In particular, at a 12-month follow-up visit, females’ general evaluation of their asthma was much worse than that of boys, even when their symptoms improved after starting omalizumab medication. Omalizumab treatment resulted in a better response in females than in males, but its safety and tolerability did not seem not gender-specific. Omalizumab probably has an effect on sex hormones directly or indirectly through the downstream processes they mediate.

It is known that pain varies across the sexes, but how it translates to practical pain management is not. Barrachina et al. compared the analgesic response of tapentadol (TAP) and oxycodone/naloxone (OXN) between sexes, in contrast to other opioids (OPO) that are frequently recommended for chronic non-cancer pain (CNCP) [46]. Enrolling 571 patients, they observed a significant gender disparity, with a higher hospital frequency and lower female tolerability, particularly in the OXN group. In this instance, compared to OXN men, females demonstrated higher hospital utilization, necessitating a higher morphine equivalent daily dose. Across all opioid groups, CNCP women were three years older than men, used benzodiazepines at a significantly higher rate, experienced more constipation and headaches as adverse events, and were more likely to report sexual dysfunction and losing interest. Women were also significantly older than men. Sex disparities were shown to be associated with higher hospital resources and worse drug acceptability in female OXN users. Also, Escorial and colleagues investigated the possible sex-related disparities among outpatients with CNCP, with an observational cross-sectional study on 806 patients [47]. Of these patients, 137 had an opioid use disorder diagnosis; the other 669 were considered as the control group. In comparison to the male controls, the female controls were older, underwent less rigorous pain therapy, and had more psychiatric prescriptions and visits. Comparing the patients to controls, the results showed lower age, greater job impairment, double the daily dose of morphine equivalent, and usage of benzodiazepines. Moreover, males reported the highest number of adverse drug responses and exhibited a higher usage of fentanyl, whereas females demonstrated a higher rate of substance use disorder and lower rate of tramadol use.

In the field of cancer treatment and related toxicity, in our previous study, we highlighted the variations in lipid profiles according to sex in patients who underwent radical surgery for adrenocortical cancer while receiving adjuvant mitotane treatment. The results showed that the lipid profiles of men and women, as well as pre- and postmenopausal women, differed. In light of the effects of mitotane on lipid levels, higher drug concentrations were associated with higher levels of HDL in all groups, total cholesterol in both males and females, triglycerides in postmenopausal females, and LDL in male patients. An increase in mitotane active metabolite (o,p’-DDE) correlated positively with HDL levels and negatively with LDL in all the evaluated groups. Additionally, increases in o,p’-DDE were positively correlated with total cholesterol in pre- and postmenopausal women and with triglycerides in premenopausal females. Considering another type of cancer, cholangiocellular adenocarcinoma, palliative chemotherapy is still the only available treatment. To evaluate the effectiveness of chemotherapy, Jordens and colleagues assessed bone mineral density (BMD) as a predictive tool [48]. Their study included 75 patients who received treatment, and they observed that BMD declined with age but that there was no difference between men and women. The predictive value of BMD was only seen in females, indicating sex-specific differences. In conclusion, BMD appears to be an independent, useful, and easily accessible predictive measure for overall survival in patients with advanced cholangiocellular adenocarcinoma. A study by Damanskiene on pediatric glioma cells evaluated the histone deacetylase inhibitor valproic acid (VPA) as an anticancer and immunomodulatory drug. They examined the impact of 0.5 and 0.75 mM VPA on the gene expressions of the co-transporters NKCC1 (*SLC12A2*), KCC2 (*SLC12A5*), and SLC5A8 (*SLC5A8*), specifically in SF8628 (girls) and PBT24 (boys) cells, using an RT-PCR technique [49]. Between the PBT24 and SF8628 controls, there was no difference in the expressions of *SLC12A2* and *SLC5A8*. Compared to the SF8628 control, the PBT24 control had substantially higher levels of *SLC12A5* expression. In PBT24, VPA treatment dramatically enhanced *SLC12A2* expression; however, SF8628 cells were unaffected. Compared to matching SF8628 groups, the PBT24-treated cells had a considerably higher level of *SLC12A5* expression. The expression of *SLC5A8* in PBT24-treated cells was noticeably higher than in the corresponding SF8628 groups. The observed differences point to possible gender-related variations in tumor cell biology. Another work conducted by Salas and colleagues observed that myometrial and *MED12* mutant leiomyoma cells repopulated cell-depleted tissue slices after 20 days of culture. They detected clusters of CD49b+ cells in tumor slices using immunofluorescence and quantitative PCR of stem cell and undifferentiated cell markers [50]. However, there were only a few CD49b+ cells found in the myometrial slices. While only a small percentage of myometrial cells were stained for this proliferation marker, almost all leiomyoma cells showed significant expression of Ki67. While the mesenchymal stem cell receptor KIT was only found in normal cells, the CD73 marker was exclusively expressed in tumor cells. Compared to myometrial cells, leiomyoma cells exhibited greater signal intensity and broader expression patterns for HMGA2 and CD24. According to the proposed research, asymmetrical division caused by the activation of CD49b+ stem cells in the myometrium results in the development of transit-amplifying KIT+ cells, which subsequently differentiate into smooth muscle cells. Activated leiomyoma CD49b+ cells, on the other hand, divide symmetrically to create clusters of stem cells that can proliferate and develop into smooth muscle cells. In conclusion, they speculated that both normal and mutant stem cells may multiply and differentiate in an organ culture over an extended period of time, providing a useful platform for the development of new therapeutics.

Studying the gender differences found during the COVID-19 epidemic is essential, and analyzing the positive and negative effects of medications on sex as well as their effectiveness in clinical trials is crucial for treating COVID-19 [51]. One possible medication that may be developed to stop the spread and complications of SARS-CoV-2 infection is VPA, a histone deacetylase inhibitor. Preclinical studies by Stakistaitis indicate that the pharmacological effects of VPA may be relevant to the pathogenetic pathways of COVID-19. Through mechanisms that may be connected to sex, VPA lowers inflammatory tissue and organ damage, suppresses the pro-inflammatory immune cell and cytokine response to infection, and prevents the entry of the SARS-CoV-2 virus. The potential benefits of VPA for treating COVID-19 include its antithrombotic, antiplatelet, anti-inflammatory, immunomodulatory, and blood serum effects on glucose and testosterone levels.

There are significant sex variations in the etiology, epidemiology, and prognosis of cardiovascular diseases (CVDs) between men and women, which is also the case when they are linked to ambient particulate matter (PM) exposure. A study conducted by Liu and colleagues on male and female C57BL/6 mice (8–10 weeks) reported that intranasal PM exposure dramatically reduced the numbers of endothelial progenitor cells (EPCs) in both blood and bone marrow, with an increase in reactive oxygen species (ROS) production in men but not in females, and males had higher serum levels of IL-6 and IL-1 than females. After being exposed to PM, males’ pulmonary expression of the antioxidant enzyme SOD1 dramatically decreased, but females’ did not. Therefore, the authors suggested that male mice’s lower expression of pulmonary SOD1 may be the cause of the PM exposure-induced drop in the EPC population [52].

Due to increased knowledge and accessibility to healthcare facilities, the gender-diverse and transgender community is a small patient group that is being seen in clinical settings more frequently [53]. Gender-affirming therapy, which involves both hormone medication and surgical intervention, typically results in partial impairment to full removal of reproductive potential and outcomes depending on the treatment modality. Prior to undergoing a medical or surgical gender transformation, transgender individuals should receive fertility preservation counseling, even though this is rarely implemented in the professional setting. Data and clinical information on transgender people’s ability to preserve their fertility are rather scarce. Despite the fact that there are several choices for fertility preservation, there are significant obstacles for the transgender population because of a lack of information and a clinical narrative that is unfamiliar to doctors and other healthcare professionals. More knowledge of this clinical agenda and the required procedures will eventually lead to far more specialized and thorough care for transgender patients who are desperately in need of fertility preservation treatments or counseling.

In conclusion, though specialized studies aimed at addressing gender inequalities are rare, gender disparities are becoming more apparent in every aspect of medicine, including drug therapy [45]. One essential variable that cannot be overlooked is sex. Gender pharmacology must always be considered while adjusting therapy in order to increase drug safety and effectiveness. Understanding gender differences encourages more health protection for both genders as well as the right use of medicines. In order to uncover potential hazards and benefits that can differ between genders, further gender study would enable reports on differences in the assimilation and response of the female organism compared to the male. Moreover, a deeper comprehension of the effects of sex and gender on pharmacological activity might facilitate the creation of customized, “tailor-made” medications.
